# Low Back Pain in Operating Room Nurses and Its Associated Factors

**DOI:** 10.12669/pjms.36.6.2836

**Published:** 2020

**Authors:** Ikbal Cavdar, Ahmet Karaman, Yasemin Ozhanli, Ayfer Ozbas

**Affiliations:** 1 Ikbal Cavdar, MSc, PhD, Professor, Surgical Nursing Department, Florence Nightingale Faculty of Nursing, Istanbul University-Cerrahpasa, Istanbul, Turkey; 2 Ahmet Karaman, MSc, PhD, Surgical Nursing Department, Florence Nightingale Faculty of Nursing, Istanbul University-Cerrahpasa, Istanbul, Turkey; 3 Yasemin Ozhanli, MSc, PhD, Surgical Nursing Department, Florence Nightingale Faculty of Nursing, Istanbul University-Cerrahpasa, Istanbul, Turkey; 4 Ayfer Ozbas, MSc, PhD, Professor, Surgical Nursing Department, Florence Nightingale Faculty of Nursing, Istanbul University-Cerrahpasa, Istanbul, Turkey

**Keywords:** Low back pain, Occupational health, Operating room nurse, Risk factors

## Abstract

**Objective:**

To determine the presence of low back pain and the associated factors in operating room nurses.

**Methods:**

The population of the descriptive study consists of 133 operating room nurses working in the operating rooms of five major hospitals located in Istanbul, and the study sample consists of 96 operating room nurses who are not on leave or sick leave between July-2016 to February 2017. Data were collected via a question form prepared by the researchers.

**Results:**

It was determined that more than half of the operating room nurses forming the sample group had low back pain and that it is affected from the practices of operating room nurses during a shift, which may cause physical strain such as year of working as an operating room nurse, bending and staying in the same position for a long time, holding an instrument for a long time, rotational movement inadequate to body mechanics, lifting/carrying heavy medical items and pushing/pulling heavy medical equipment.

**Conclusion:**

Majority of operating room nurses had low back pain and it was associated with coercive movements during surgery.

## INTRODUCTION

Anatomical, physiological, psychological and socio-cultural characteristics of healthcare professionals, along with working technique and physical characteristics of the working environment, negatively affect the musculoskeletal system. The prevalence of musculoskeletal system disorders is 5.7% in industrial workers while this rate increases to 8.8% in people working in hospitals.^1^

Nurses, who have an important role among healthcare professionals, experience musculoskeletal system disorders, including upper back, neck, shoulder and joint pain. The most common musculoskeletal system problem observed in nurses is low back pain.^2^,^3^ Different studies in the literature evaluated one-year incidence of low back pain in nurses in various countries and found this rate to be 56.8% in China, 40.6% in Hong Kong, 59% in Japan, 50.1% in Tunisia and 40-50% in the United States of America.^4^ Even though there is no research in Turkey that assesses the rate of low back pain in nurses in a way that can be generalized to all nurses, there are studies performed with certain groups. Gul et al. (2014) found that 66.4% of nurses experience low back pain while Yılmaz and Özkan (2008) found that 39.9% of nurses had low back pain.^3^,^5^

Nurses working in the operating room possess a greater risk in terms of musculoskeletal problems due to pulling and pushing gurneys, beds and other equipment, transferring the patient to the operating table or gurney, supporting an extremity for a long time and standing in the same position for a long time.^2^,^3^

Keeping in mind those risk factors about musculoskeletal problems in operating room nurses will increase workforce loss and costs by causing functional insufficiencies in the future, determining the risk factors for low back pain in operating room nurses and taking the necessary precautions are of vital importance. The study was conducted in order to determine the rate of low back pain in operating room nurses and its associated factors.

## METHODS

The population of this descriptive study consisted of 133 nurses who worked in the operating rooms of five major hospitals in Istanbul. Rather than sampling, census was aimed. Inclusion criteria were determined as being working in the operating rooms of the study hospitals for at least six months, not being on leave/sick leave between July 2016 and February 2017 and volunteering to participate in the study. Because some nurses had taken a leave in the defined time period and some nurses declined to participate, 72.1% of the universe was reached and the study was completed with 96 operating room nurses.

Data were collected using a “Data Collecting Form” developed by the researchers in the light of the literature.^2^,^3^,^5^-^9^ The form consisted of four sections that questioned descriptive characteristics of the nurses, working conditions, the frequency of practices that may cause physical strain during work and the characteristics of low back pain encountered in operating room nurses. Data were collected using face-to-face interviewing technique on working days of operating room nurses and in time periods that won’t interfere with the work order.

The data obtained from the study was analyzed with the Statistical Package for Social Science 21.0 packaged software. Frequency, percentage, mean, standard deviation, Chi-squared and student’s t test were used when appropriate. Results were evaluated with 95% confidence interval and p<0.05 significance level.

Written approval from a faculty of medicine clinical research ethics committee (Approval Date and Number: 12.07.2016-A-24) and hospitals that the study was conducted in were obtained prior to the start of the study. Verbal and written consent was also obtained from nurses before data collecting tools were applied.

## RESULTS

Out of operating room nurses that participated in the study, 52.2% were between 21-35 years of age. 82.3% were female. 53.1% were married. 55.2% had a graduate degree and 47.9% had 11 years or more professional experience and 37.5% had been working has an operating room nurse for one to five years. About 72.9% did not smoke; 84.4% worked out regularly and 63.5% defined their health status as “well”. About 43.8% had a responsibility for caring for a relative and/or children or that required carrying weight outside working hours. 71.9% had normal BMI (18.5 kg/m^2^<BMI< 25.0 kg/m^2^). Total time spent standing in one shift was found to be 6.5208±1.56931 hours. 76% of operating room nurses took a break during work and time worked until a break was found to be 4.0411±1.16756 hours.

Almost 28.1% of operating room nurses positioned a patient one to two times in one shift. 31.3% moved the patient from operating table to gurney one to two times in one shift. 56.3% of nurse leaned more than five times in one shift. 67.7% stood in the same position for a long time more than five times in one shift. 50.0% held equipment for a long time more than five times in one shift. 33.3% supported any part of patient for a long time one to two times in one shift. 31.3% rotated backwards more than five times in one shift, 32.3% of operating room nurses lifted/carried heavy medical equipment more than five times in one shift, while 34.4% pulled/pushed heavy medical equipment more than five times in one shift.

In this study 67.7% of operating room nurses who participated had low back pain at any time. 93.8% of those experienced low back pain in the past year, 87.7% experienced low back pain in the current year while 55.4% experienced low back pain on the day of the interview.

The prevalence of low back pain was higher in nurses who worked as an operating room nurse for six to 10 years than others who worked for less than one year, one to five years and more than 11 years and the difference was significant (p<0.05). Also, the prevalence of low back pain was significantly higher in operating room nurses with normal BMI when compared to nurses with BMI<18.5 kg/m^2^ and 25.0 kg/m^2^<BMI< 30.0kg/m^2^ (p<0.05) (Table-I).

**Table-I:** Comparison of the Frequency of Low Back Pain in Operating Room Nurses with Descriptive Characteristics and Working Conditions.

Characteristics	Low Back Pain Present (n=65)		Low Back Pain Absent (n=31)		X^2^/t	p

	n	%	n	%		
Age (years)					0.016^a^	0.898
21 to 35	36	69.2	16	30.8		
36 and older	29	65.9	15	34.1		
Marital Status					0.203^a^	0.503
Married	33	64.7	18	35.3		
Single	32	71.1	13	28.9		
Total Time Worked as an Operating Room Nurse					11.450^a^	0.010*
Less than 1 year	1	14.3	6	85.7		
1 to 5 years	27	75.0	9	25.0		
6 to 10 years	18	78.3	5	21.7		
11 years and above	19	63.3	11	36.7		
Smoking					0.000	1.000
Yes	18	69.2	8	30.0		
No	47	67.1	23	32.9		
Regular Exercise					0.991^a^	0.319
Yes	8	53.3	7	46.7		
No	57	70.4	24	29.6		
Responsibility that Requires Carrying Weight or for Caring for Someone Outside Working Hours					2.042^a^	0.564
Never or rarely	31	73.8	11	26.2		
Sometimes	22	64.7	12	35.3		
Often	5	71.4	2	28.6		
Always	7	53.8	6	46.2		
BMI					7.969^a^	0.047*
Underweight (<18.5 kg/m2)	1	20.0	4	80.0		
Normal (18.5 kg/m2<BMI< 25.0 kg/m2)	51	73.9	18	26.1		
Overweight (25.0kg/m2<BMI< 30.0kg/m2)	12	57.1	9	42.9		
Obese ( BMI≥30.0kg/m2)	1	100.0	0	0.0		
Taking a break during work					0.001^a^	0.801
Yes	50	68.5	23	31.5		
No	15	65.2	8	34.8		
Total Time Spent Standing During One Shift (Hours) (Mean±Sd)	6.48±1.34		6.61±1.99		0.395^b^	0.694

a=Chi-squared test, b=Independent samples *t* test,

* =p<0.05, %=Given as line percentage, Sd: Standard deviation, BMI: Body mass index

Nurses who leaned more than five times in one shift experienced low back pain more frequently than nurses who leaned for three to five times in one shift, one to two times in one shift and never leaned. Nurses who stood in the same position for a long time three to five times in one shift had more back pain than other who stood in the same position for a long time more than five times, one to two times or never stood in the same position. Nurses who held equipment for a long time three to five times in one shift had more back pain than other who held equipment for a long time more than five times, one to two times or never held equipment for a long time. Finally, nurses who rotated backwards more than 5 times in one shift experienced low back pain more frequently than nurses who rotated backwards for 3 to 5 times in one shift, one to two times in one shift and never rotated. All differences were statistically significant (p<0.05) (Table-II).

**Table-II:** Comparison of the Frequency of Low Back Pain in Operating Room Nurses with Practices that may Cause Physical Strain.

Expressions	Low Back Pain Present (n=65)		Low Back Pain Absent (n=31)		X^2^	p

	n	%	n	%		
Positioning a patient					2.787	0.426
More than 5 times a day	8	72.7	3	27.3		
3 to 5 times a day	15	75.0	5	25.0		
1 to 2 times a day	20	74.1	7	25.9		
Never	22	57.9	16	42.1		
Moving the patient from operating table to gurney					3.708	0.295
More than 5 times a day	7	63.6	4	36.4		
3 to 5 times a day	13	86.7	2	13.3		
1 to 2 times a day	21	70.0	9	30.0		
Never	24	60.0	16	40.0		
Leaning					15.078	0.002*
More than 5 times a day	42	77.8	12	22.2		
3 to 5 times a day	16	76.2	5	23.8		
1 to 2 times a day	6	37.5	10	62.5		
Never	1	20.0	4	80.0		
Standing in the same position for a long time					17.661	0.001*
More than 5 times a day	49	75.4	16	24.6		
3 to 5 times a day	12	80.0	3	20.0		
1 to 2 times a day	2	16.7	10	83.3		
Never	2	50.0	2	50.0		
Holding equipment for a long time					12.898	0.005*
More than 5 times a day	37	77.1	11	22.9		
3 to 5 times a day	17	81.0	4	19.0		
1 to 2 times a day	8	38.1	13	61.9		
Never	3	50.0	3	50.0		
Supporting any part of patient for a long time					2.644	0.450
More than 5 times a day	14	73.7	5	26.3		
3 to 5 times a day	19	73.1	7	26.9		
1 to 2 times a day	22	68.8	10	31.3		
Never	10	52.6	9	47.4		
Rotating backwards					14.953	0.002*
More than 5 times a day	24	80.0	6	20.0		
3 to 5 times a day	23	76.7	7	23.3		
1 to 2 times a day	17	60.7	11	39.3		
Never	1	12.5	7	87.5		
Lifting/carrying heavy medical equipment					10.685	0.014*
More than 5 times a day	24	77.4	7	22.6		
3 to 5 times a day	18	78.3	5	21.7		
1 to 2 times a day	19	65.5	10	34.5		
Never	4	30.8	9	69.2		
Pulling/pushing heavy medical equipment					10.862	0.012*
More than 5 times a day	26	78.8	7	21.2		
3 to 5 times a day	19	73.1	7	26.9		
1 to 2 times a day	18	64.3	10	35.7		
Never	2	22.2	7	77.8		

*X^2^*= Chi-squared test,

* =p<0.05, %= given as line percentage

Similarly, operating room nurses who lifted/carried heavy medical equipment 3 to 5 times in one shift had more back pain than other who stood in the same position for a long time more than 5 times, 1 to 2 times or never stood in the same position. Also, nurses who pulled/pushed heavy medical equipment more than 5 times in one shift experienced low back pain more frequently than nurses who pulled/pushed heavy medical equipment for three to five times in one shift one to two times in one shift and never pulled/pushed. These differences were statistically significant as well (p<0.05) (Table-II).

## DISCUSSION

Low back pain is one of the most common musculoskeletal problems observed among nurses.^10^,^11^ Different studies with nurses and healthcare professionals revealed that more than half of the participants experienced low back pain.^2^,^3^,^5^,^6^ Similar results were obtained with studies from China, Tunisia, the United Kingdom, Sweden, Iran, Italy, Hong Kong, Nigeria and Japan.^4^ Likewise, more than half of the nurses in the present study expressed having low back pain.

Age is among the individual risk factors that may cause low back pain. There was no statistically significant association between age and the prevalence of low back pain in this study. Even though there are studies in the literature indicating that age variable does not impact the prevalence of low back pain^9^,^12^,^13^, studies implicating the contrary are also present.^6^,^10^,^11^

Marital status variable did not cause a difference in the frequency of low back pain in the present study. Despite there are studies, which found that single individuals experience more low back pain than married individuals^2^,^14^, other studies support our findings.^3^,^6^,^15^

As the years worked increase, total exposure to risks in the workplace also increase, increasing the risk of associated health deterioration. In the present study, individuals who had been working for an operating room nurse were found to have more low back pain. Even though many studies performed with healthcare professionals^2^,^3^,^15^ yielded similar results, some studies have reported that years worked don’t affect the frequency of low back pain.^9^ Considering that as the years worked increase, total exposure to risks that may cause musculoskeletal problems also increase, our study results are expected.

Smoking is reported to be a risk factor that increases the incidence of low back pain.^16^ In the present study, smokers had more low back pain than nonsmokers, yet the difference did not reach statistical significance. Karahan et al^8^, Daraiseh et al^17^, Vieira et al^18^ reported that smokers experienced low back pain more frequently and the difference was significant. Similar to our study, there are certain studies that found no statistically significant difference among smokers and non-smokers in terms of the frequency of low back pain but smokers experience more low back pain.^2^,^15^ Due to adverse effects of smoking on vascular system, blood circulation in spine and muscles are also negatively affected, making the musculoskeletal system more susceptible to external factors.

Regular exercise affects the individual positively both physically and mentally. It can especially prevent the development of musculoskeletal problems by strengthening the muscles. Similar to our study, Çil-Akıncı et al^2^, Aksakal et al^6^, Yılmaz and Özkan^3^, Vieira, et al^18^ also found that nurses who exercised regularly experienced less low back pain.

Body mass index another individual factor that impacts the musculoskeletal system. In the present study, nurses with normal BMI were found to have more low back pain than overweight nurses. Even though Daraiseh et al^17^, Heuch et al^19^, Shiri et al^20^ reported that individuals with high BMI had higher incidence of low back pain, there are also studies^6^,^9^,^13^ that BMI causes no difference.

Repetitive movement, inappropriate posture and excessive force exertion are important factors causing musculoskeletal problems.^16^ In the present study, nurses who performed practices that may cause physical strain, including leaning, standing the same position for a long time, holding equipment for a long time, rotating backwards, lifting/carrying heavy medical equipment and pulling/pushing heavy medical equipment, more often in one shift experienced significantly more low back pain. Similarly, results of Çil-Akıncı et al^2^, Aksakal et al^6^, Alexopoulos et al^7^, Karahan et al^8^, Dlungwane et al^12^ are in concordance with our findings.

## CONCLUSIONS

In conclusion, it is recommended that operating room nurses should be trained about body mechanics, necessary steps should be taken to ensure adequate number of nurses in order to minimize the frequency of practices that may cause physical stress, acceptable break time in a shift should be provided to nurses when increasing the number of nurses is not possible, operating rooms should be arranged ergonomically and regular exercise programs should be organized to increase the endurance of lumbar muscles of operating room nurses.

### Note:

This study was presented (poster presentation) at ‘‘8th EORNA Congress’’ 4-7 May 2017, in Rhodes, GREECE.

### Authors Contribution:

**IC, AK, YO, AO:** Conceived, designed and did statistical analysis & editing of manuscript.

**IC, AK, YO, AO:** Did data collection and manuscript writing.

**IC, AK, YO, AO:** Did review, final approval of manuscript and are responsible for integrity of study.
